# Long-Term Survivability of Tardigrade *Paramacrobiotus experimentalis* (Eutardigrada) at Increased Magnesium Perchlorate Levels: Implications for Astrobiological Research

**DOI:** 10.3390/life14030335

**Published:** 2024-03-04

**Authors:** Paulina Anna Wilanowska, Piotr Rzymski, Łukasz Kaczmarek

**Affiliations:** 1 Department of Animal Taxonomy and Ecology, Faculty of Biology, Adam Mickiewicz University in Poznań, 61-614 Poznań, Poland; pauwil3@amu.edu.pl; 2 Department of Environmental Medicine, Poznan University of Medical Sciences, 60-806 Poznań, Poland; rzymskipiotr@ump.edu.pl

**Keywords:** extremophiles, Mars, Tardigrada, water bears

## Abstract

Perchlorate salts, including magnesium perchlorate, are highly toxic compounds that occur on Mars at levels far surpassing those on Earth and pose a significant challenge to the survival of life on this planet. Tardigrades are commonly known for their extraordinary resistance to extreme environmental conditions and are considered model organisms for space and astrobiological research. However, their long-term tolerance to perchlorate salts has not been the subject of any previous studies. Therefore, the present study aimed to assess whether the tardigrade species *Paramacrobiotus experimentalis* can survive and grow in an environment contaminated with high levels of magnesium perchlorates (0.25–1.0%, 1.5–6.0 mM ClO_4_^−^ ions). The survival rate of tardigrades decreased with an increase in the concentration of the perchlorate solutions and varied from 83.3% (0.10% concentration) to 20.8% (0.25% concentration) over the course of 56 days of exposure. Tardigrades exposed to 0.15–0.25% magnesium perchlorate revealed significantly decreased body length. Our study indicates that tardigrades can survive and grow in relatively high concentrations of magnesium perchlorates, largely exceeding perchlorate levels observed naturally on Earth, indicating their potential use in Martian experiments.

## 1. Introduction

Astrobiology is an interdisciplinary branch of science that combines fields such as astronomy, biology, physics, chemistry, geology, planetology, and even ethics. Its main aims include the origin of life, the abiotic conditions necessary for Earth organisms to survive, searching for life on other planets and moons, and many others. Therefore, astrobiological research is diverse and raises many difficult questions, such as “What is life?”. Such subjects appeared for the first time in the works of ancient philosophers such as Aristotle or Epicurus [1,2].

Thanks to the constantly developing detection methods, more and more exoplanets are being discovered (at present, more than 5000; see http://exoplanet.eu/catalog/ (accessed on 27 February 2024)). Scientists are working on methods to determine whether or not life can exist on a given planet. Examples of attempts to estimate the survival chances of terrestrial organisms on other planets include the ATI (Active Tardigrade Index) and CTI (Cryptobiotic Tardigrade Index). They are used to determine the potential survival of tardigrades in an active state and cryptobiosis on water–rocky and water–gas planets. In this approach, six physical parameters are taken into account: the radius of the planet; its density, escape velocity, rotation period, and temperature; and the pressure on the surface of the planet [3]. However, other conditions, such as high levels of reactive chemicals, may prevent life survival. When perchlorates, in the form of salts (e.g., magnesium, sodium, calcium perchlorates) were found to be ubiquitously in Martian regolith at levels far exceeding those noted on Earth [4,5,6,7,8,9], the possibility of life emergence on this planet was challenged due to their toxicity [10,11,12]. According to on-site studies performed in the selected Martian regions, e.g., near the Gale crater, the average level of perchlorates was 0.6%, not excluding the possibility that lower and higher levels may characterize some regions. In fact, reinterpreting the results obtained by the Viking Lander suggests that perchlorate levels may, in some locations, reach up to 0.1% [13].

On the other hand, the presence of perchlorates could possibly be overcome by extremophiles or extremotolerant organisms, as they possess unique biological features responsible for surviving under particular stressors, often acting at levels surpassing those observed naturally on Earth [14]. However, some soil bacteria and cyanobacteria have been demonstrated to maintain growth under increased perchlorate exposure due to their enzymatic reduction (e.g., *Azospirillum* spp., *Dechloromonas* spp., and *Dechlorosoma* spp.) [15] or protection from perchlorate-induced oxidative damage (e.g., representatives of *Chroococcidiopsis*, *Leptolyngbya*, *Arthronema*, *Geitlerinema,* and *Cephalothrix* genera) [16,17]. However, it is unknown whether extremotolerant animals could also survive perchlorate stress at levels expected on Mars. 

In general, perchlorates are well known to be toxic to vertebrates and invertebrates, e.g., *Caenorhabditis elegans* (Maupas, 1900) at levels as low as 0.00005% [18] or to *Daphnia magna* (Straus, 1820) at 0.05% [19]. However, selected invertebrates are known for their resistance to various extreme conditions [20,21]. Among them, tardigrades, free-living microinvertebrates associated with terrestrial and aquatic environments, stand out to the point where they have gained the popular title of the “toughest animals on Earth” [22,23]. So far, more than 1400 species of Tardigrada (commonly called water bears) have been described worldwide [24]. Tardigrades inhabit most terrestrial and aquatic (freshwater and marine) environments. They can be found in mosses, lichens, soil, leaf litter, sediments, and aquatic plants [23]. Some tardigrade species can resist space vacuum, high-ionizing and UV radiation levels, very low and high temperatures, low and high atmospheric pressure, varying chemicals, or even a lack of oxygen. Their extraordinary abilities to withstand different types of extremities are primarily due to cryptobiosis, particularly anhydrobiosis induced by desiccation. (e.g., [25,26,27,28]). Anhydrobiosis (from the Greek for “life without water”) results in almost a complete loss of body water and entering a state of reversible suspension [29,30]. Anhydrobiosis is very important for survival in extreme environments with a periodic lack of water, which can affect growth and reproduction. It also affects lifespan and may, therefore, slow down the rate of evolution [29]. Anhydrobiosis in tardigrades is a complex phenomenon. It includes entering, permanent, and leaving steps, which correspond to dehydration (tun formation), tun state (desiccated state), and rehydration, respectively [31]. These steps are well known at the level of the organism’s morphology [32], but a full understanding of their mechanisms requires the consideration of additional factors. It is known that the survival rate of anhydrobiosis can be affected by the type of environment (terrestrial, freshwater, etc.), feeding behavior (diet), environmental/culture conditions (e.g., ambient temperature, water quality, culture substratum), and other factors such as body size, conditions of dehydration, and the number and duration of anhydrobiosis episodes [33]. These unique abilities are facilitated by different metabolites produced by tardigrades, mainly different types of proteins (but not only), including cytoplasmic-abundant heat-soluble (CAHS) proteins, cytotardin, mitochondrial-abundant heat-soluble (MAHS) proteins, secretory-abundant heat-soluble (SAHS) proteins, late embryogenesis-abundant (LEA) proteins, damage suppressor protein (Dsup), heat shock proteins (Hsps), and glutathione peroxidase (GPx) (for a review, see [34]). Due to their resistance and excellent ability to enter into anhydrobiosis, tardigrades are considered suitable for space studies and astrobiological research [28,35,36,37].

Whether tardigrades can tolerate perchlorate salts at the existing levels on Mars remains unknown. Considering hypothetically that such tolerance exists in tardigrades, further studies on the identification of pathways behind it would be motivated to test whether these pathways could eventually be translated into enhancing the resistance of other organisms useful for Mars exploration.

Previous research has already indicated that some species, i.e., *Paramacrobiotus experimentalis* Kaczmarek, Mioduchowska, Poprawa & Roszkowska, 2020, can survive short-term exposure (24 h) to magnesium perchlorate, which is a strong oxidizer [38], suggesting that they may possess features to withstand perchlorate-induced stress. However, since perchlorates are continuously present in Martian regolith at elevated levels, any potential on-site application of tardigrades would require long-term survival in a perchlorate-contaminated environment. Such survival has never been studied before. Therefore, this research aimed to evaluate whether tardigrades of the species *Pam. experimentalis* can withstand and grow for eight weeks in an environment contaminated by 0.25–1.0% magnesium perchlorate, which are levels that can be expected in Martian regolith. 

## 2. Materials and Methods

### 2.1. Culturing Procedure

Specimens of *Pam. experimentalis* were initially collected from terrestrial mosses in Madagascar in 2013 [39] and continuously cultured at the Department of Animal Taxonomy and Ecology (Faculty of Biology, Adam Mickiewicz University in Poznań, Poland). Eggs of *Pam. experimentalis* were extracted from the stock culture, and a new culture dedicated to the experiment was established. Specimens were cultured for six months according to Roszkowska et al. [37] in order to obtain the number of individuals necessary to start the experiment. In summary, the specimens were kept in plastic Petri dishes (⌀ 55 mm), scratched on the bottom with sandpaper, and filled with culture medium containing 75% distilled water mixed with 25% still spring water Żywiec Zdrój (Cięcina, Poland). The spring water contained bicarbonates (121.06 mg/L), fluorides (0.07 mg/L), Mg^2+^ (5.37 mg/ L), Ca^2+^ (36.39 mg/L), and Na^+^ (7.79 mg/L) and had a pH of 7.4 [40]. The culture medium was changed every seven days. Such cultures were stored in an environmental chamber (POL EKO KK115 TOP+, POL-EKO Aparatura, Wodzisław-Śląski, Poland) in complete darkness and at a temperature of 18 °C. Tardigrades were fed once a week with rotifers *Lecane inermis* (Bryce, 1892) and nematodes *Caenorhabditis elegans* (added ad libitum).

### 2.2. Experimental Design

Freshly hatched 1–2-day-old juvenile specimens were selected for the experiment and transferred with an automatic pipette to a separate 24-well culture plate (Nest Scientific Biotechnology, GenoPlast, Rokocin, Poland) with the use of a stereomicroscope Olympus SZX7 (Olympus, Tokyo, Japan). The wells were filled with 2 mL of the magnesium perchlorate (Sigma-Aldrich, Darmstadt, Germany) solution at the following concentrations: 0.1%, 0.15%, 0.2%, and 0.25%. All magnesium perchlorate solutions were prepared using distilled water (electric conductivity: 0.46 μS/cm; pH = 7.0). A total of 24 specimens were selected for each experiment and control, i.e., 120 specimens in total. Each tardigrade was cultured separately in the individual well, i.e., for each perchlorate concentration, there were 24 repetitions and another 24 for the control. The individuals were fed weekly with rotifers and nematodes added ad libitum (as described above for stock cultures). The medium was changed once per week, and dead specimens (considered dead when not moving at more than 24 h after being put into the clean water) were removed from the experiment. In such conditions, tardigrades were cultured for 56 days in total. Culture plates with tardigrades were kept for the entire experiment in an environmental chamber in the dark at 18 °C and 40% relative humidity.

### 2.3. Microscopy Measurements

At the end of the experiment, permanent microscopic slides (one tardigrade individual per slide) were prepared in Hoyer’s medium. After preparation, all slides were placed in an incubator at 56 °C for 24 h. The microscope slides were secured with nail polish before microscope examination. Specimens were examined and measured using a Phase-Contrast Microscope (Olympus BX41) associated with the Olympus SC50 photo camera and Olympus Cell Sens Entry software v. 3.2. Body length was measured from the anterior extremity to the end of the body, excluding the hind legs.

### 2.4. Statistical Analysis

The mortality of the specimens was calculated based on the number of specimens that survived in each experiment with a comparison to the control. The differences in body length between the control and exposed groups were analyzed using a non-parametric Kruskal–Wallis ANOVA (due to some data not meeting the assumption of Gaussian distribution) with a post hoc Dunn’s test. All statistical analyses were performed using the Statistica v. 13 package (StatSoft, Tulsa, OK, USA), and GraphPad Prism 5.1 package (GraphPad Software, Inc., La Jolla, CA, USA) was employed to prepare graphical presentations of the results. A *p*-value below 0.05 was deemed statistically significant.

## 3. Results

**Figure 1:**
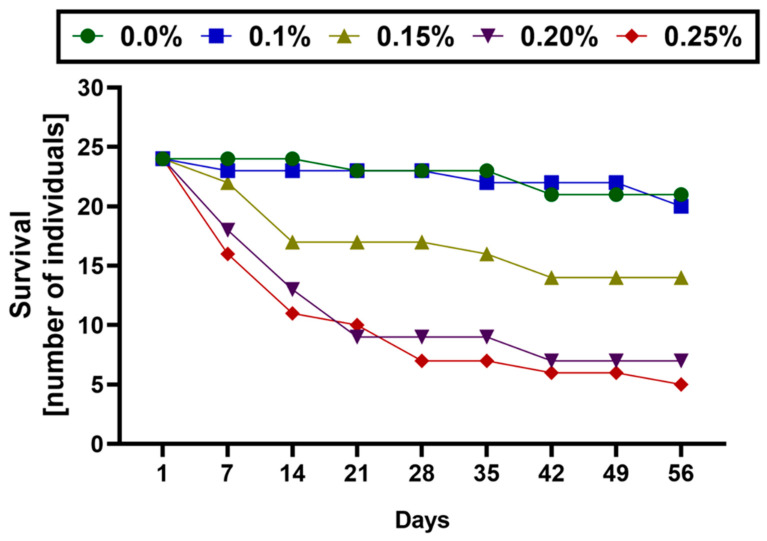
Survivability of the *Pam. experimentalis* of the experiments in different magnesium perchlorate concentrations and control.

**Figure 2:**
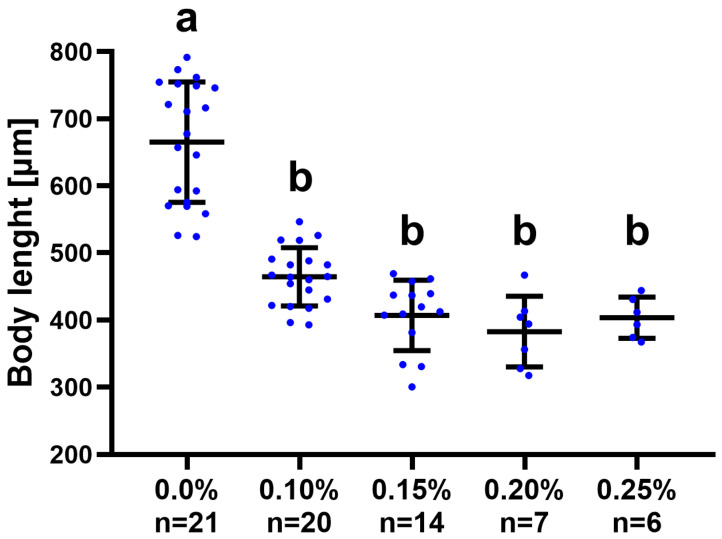
Differences in body length of the *Pam. experimentalis* in the control group and groups exposed to different magnesium perchlorate concentrations (0.1–0.25%). Blue points represent the body length of the individual specimens in each group, the horizontal line represents the mean, and the whiskers represent the standard deviation. Different letters above the columns denote statistically significant differences (*p* < 0.05) of pairwise post hoc comparisons (Dunn’s test following Kruskal–Wallis ANOVA).

The survival rate of tardigrades decreased with an increase in the concentration of the magnesium perchlorate. The number of surviving individuals and general survivability in the following weeks of the experiment are presented in Table 1 and Figure 1. At the 0.10% magnesium perchlorate level, the survivability rate was similar to the control, while it decreased rapidly starting from the 0.15% solution, with only one-fifth of the specimens surviving at a 0.25% magnesium perchlorate concentration. The curve showing the number of dying individuals was steeper during the first two weeks, which indicates a higher mortality rate in the initial stage of the experiment (Figure 1). Body lengths of all specimens that survived to the end of the experiment in each group are presented in Figure 2 and provided in the Supplementary Materials (Table S1). The body length of the tardigrade specimens exposed to all magnesium perchlorate concentrations differed significantly from the control group, but not from each other (Figure 2).

## 4. Discussion

The present study explores the response of tardigrade *Pam. experimentalis* to long-term magnesium perchlorate exposure at concentrations that are in the range of those noted in Martian regolith and exceed by 1000-fold the highest level noted naturally on Earth in evaporites in hyperarid regions (0.00025% wt) and by 100-fold the highest level found in superficially running waters [10,41,42,43,44]. Therefore, it is evident that no organism living on Earth, including tardigrades, could be naturally challenged by the conditions applied in the present study to evolve specific adaptation mechanisms of magnesium perchlorate resistance.

Although the magnesium perchlorate solution was toxic to the tested tardigrade species at levels exceeding 0.1%, members of the tested tardigrade species survived at a 21% rate for as long as eight weeks. For comparison, *Bacillus subtilis* (Ehrenberg, 1835) exposed to perchlorate at levels expected at Mars revealed a significant decrease in viability after 30–60 s of exposure [45]. These findings demonstrate that the studied tardigrade species possess some unique abilities, advocating for further exploration on the molecular and biochemical level and predisposing *Pam. experimentalis* as a model species for Martian research. Importantly, it cannot be excluded that the toxicity observed in the present study partially resulted from the elevated magnesium concentration and not only from the action of perchlorate ion, as elevated levels of this element have been shown to affect freshwater invertebrates adversely [46,47]. Nevertheless, Martian regolith contains perchlorate in the form of salts, including magnesium perchlorate [4,6]. Therefore, an organism that would be exposed to them would have to resist the increased level of perchlorate anion as well as component cation. With this in mind, the present study did not aim to evaluate the tolerance of tardigrades to magnesium, but aimed to evaluate that to magnesium perchlorate, because this is the compound actually present in Martian regolith. Nevertheless, it has been accepted that the toxicity of perchlorate salts mostly results from their dissolution and release of perchlorate anion, which has oxidizing properties and is exceedingly mobile and persistent due to high activation energy related to its reduction [48].

It is plausible that tardigrade’s tolerance to magnesium perchlorate is a by-product of their ability to enter cryptobiosis and withstand desiccation. Both desiccation and perchlorate stresses are comparable regarding reducing cellular water potential and inducing oxidative stress [49]. An increased generation of reactive oxygen species can eventually lead to reactions with proteins, lipids, and nucleic acids and, consequently, cause damage to these molecules, resulting in protein carbonylation, lipid peroxidation, the oxidation of pyrimidine and purine bases, the formation of nucleobase radicals and, ultimately, DNA breaks [50,51,52]. However, tardigrades possess various protective molecules, including damage suppressor protein (Dsup) and Dsup-like proteins, cytotardin, cytoplasmic-abundant heat-soluble proteins, secretory-abundant heat-soluble proteins, mitochondrial-abundant heat-soluble proteins, and late-embryogenesis proteins [34,53,54,55,56,57,58,59]. Therefore, it can be postulated that these molecules are also involved in attenuating the stress which is induced over the course of perchlorate exposure. Of note, adding magnesium perchlorate increases the electric conductivity of the solution [60], while some tardigrades are known for their extraordinary osmoregulation that could support the withstanding of high salinity [27,61,62]. However, further studies are required to elucidate the exact molecular and biochemical pathways involved in tardigrades’ tolerance to high environmental concentrations of perchlorate.

As evidenced in the present study, magnesium perchlorate exposure significantly affected the body length of the tested specimens, particularly at levels higher than 0.1%. The mechanism behind this phenomenon requires further research. Body size is an important parameter that was assessed in this study because it shows that the tardigrades grew slower under stress conditions caused by high perchlorate levels. Given that food availability was the same, this indirectly indicates the adverse effect of the environment on metabolism or behavior. Previous studies have also shown that toxin-induced stress can be associated with decreased size in some microinvertebrates (e.g., in zooplankton species [63]). Such an effect can be due to the direct inhibiting effect of chemicals on growth processes or indirectly result from behavioral changes (e.g., decreased activity and feed rate).

The present study indicates that magnesium perchlorate can be added to the list of conditions that some tardigrade species can resist, which already includes space vacuum, high-ionizing and UV radiation levels, very low and high temperatures, low and high atmospheric pressure, varying chemicals (ethanol, hydrogen sulfide, bromomethane, carbon dioxide, toxic metals), or even a lack of oxygen (e.g., [25,26,27,28,64,65]). Such resistance to extreme stresses can potentially be translated into novel technologies useful in preserving biomaterial in dry states and strategies protecting against different stressors existing on other planets, including Mars. For example, it has been shown that the discovery of the high radiotolerance of tardigrades could eventually be translated into an approach that significantly increases the tolerance to the X-ray radiation of human cells transfected to express Dsup (e.g., [34,66]). As postulated, genetic engineering and synthetic biology may advance tardigrades in various fields and translate resistance and tolerance mechanisms to practical use (e.g., in space exploration and medicine) [34,67].

This study’s limitations should be stressed. First, the tardigrades were exposed only to perchlorate, while on Mars, these chemicals interact with other conditions, which may have synergistic effects such as UV radiation [45]. On the other hand, tardigrades are also known to sustain UV exposure owing to the protective role of Dsup and efficient DNA repair [68,69]. Second, the present experiments employed perchlorate solutions without Martian regolith (samples of which, at this moment, have not been brought to Earth), while the water does not exist in the liquid phase on the Martian surface. A study employing a Martian regolith simulant indicates that if liquid water emerged on this planet, it would be rapidly enriched in various ions that could have individual and additive effects on exposed living organisms [60,70]. One could also point out that in the present study, the perchlorate salt was completely dissolved in the aqueous solution, while on Mars, they would be released from the regolith. However, the perchlorate anion has a very high solubility in water, which, coupled with its limited propensity to adsorb to most soil surfaces, leads to high mobility in aqueous environments [48], and there is no indication that it would behave in any other way in inorganic Martian regolith. Third, this research was focused on one tardigrade species which is non-challenging to culture. However, results should not be generalized over the entire phylum without further research. Fourth, in addition to the magnesium perchlorate employed in the present study, the Martian regolith was also found to contain varying levels of sodium and calcium perchlorates [4,6,71,72,73]. Fifth, the animals were exposed without preconditioning to other harsh conditions (which was shown to increase tolerance to, e.g., phosphine [74]), although this only further highlights their abilities to tolerate magnesium perchlorate.

## 5. Conclusions

The study indicates that *Pam. experimentalis* may be suitable for future astrobiological studies concerning Mars, as it showed that this species has an ability to demonstrate long-term resistance to magnesium perchlorate at levels highly exceeding those observed naturally on Earth and in the range that can be expected on Mars. These observations, along with the results of our previous studies [38], indicate that tardigrades may be suitable animal model organisms for further astrobiological research in the context of Mars. Future investigations should address the exposure of these microinvertebrates to various perchlorate salts under other simulated Martian conditions and explore whether they may be able to reproduce. Further biochemical and molecular studies are necessary to understand the exact mechanisms behind this tolerance and constitute an essential step towards postulating the translation of this mechanism for practical use in Martian exploration and protection from harmful chemicals.

## Figures and Tables

**Table 1:** The number of live specimens of the species *Pam. experimentalis* after each specific day of the experimental period in different magnesium perchlorate concentrations (ER—experimental regimes) and the survivability of the specimens at the end of the experiment.

ER/Days	1	7	14	21	28	35	42	49	56	Survivability
control	24	24	24	23	23	23	21	21	21	87.5%
0.10%	24	23	23	23	23	22	22	22	20	83.3%
0.15%	24	22	17	17	17	16	14	14	14	58.3%
0.20%	24	18	13	9	9	9	7	7	7	29.2%
0.25%	24	16	11	10	7	7	6	6	5	20.8%

## Data Availability

Data are contained within the article and Supplementary Materials.

## Supplementary Materials

The following supporting information can be downloaded at: https://www.mdpi.com/article/10.3390/life14030335/s1, Table S1: The body length of the *Pam. experimentalis* specimens in different magnesium perchlorate concentrations and control at the end of the experiment.
