# Correction: Wilanowska et al. Long-Term Survivability of Tardigrade *Paramacrobiotus experimentalis* (Eutardigrada) at Increased Magnesium Perchlorate Levels: Implications for Astrobiological Research. *Life* 2024, *14*, 335

**DOI:** 10.3390/life14050573

**Published:** 2024-04-30

**Authors:** Paulina Anna Wilanowska, Piotr Rzymski, Łukasz Kaczmarek

**Affiliations:** 1 Department of Animal Taxonomy and Ecology, Faculty of Biology, Adam Mickiewicz University in Poznań, 61-614 Poznań, Poland; pauwil3@amu.edu.pl; 2 Department of Environmental Medicine, Poznan University of Medical Sciences, 60-806 Poznań, Poland; rzymskipiotr@ump.edu.pl

There was an error in the original publication [1]. The Abstract and Introduction stated the incorrect concentration of magnesium perchlorate salt employed in the manuscript.

In the Abstract, the fourth sentence should read as follows: “Therefore, the present study aimed to assess whether the tardigrade species *Paramacrobiotus experimentalis* can survive and grow in an environment contaminated with high levels of magnesium perchlorates (0.10–0.25%, 0.6–1.5 mM ClO_4_^−^ ions)”. The last sentence of the Introduction section should read as follows: “Therefore, this research aimed to evaluate whether tardigrades of the species *Pam. experimentalis* can withstand and grow for eight weeks in an environment contaminated by 0.10–0.25% magnesium perchlorate, which are levels that can be expected in Martian regolith”.

The authors state that the scientific conclusions are unaffected. This correction was approved by the Academic Editor. The original publication has also been updated.
