# Supplementary material for: Long-Term Survivability of Tardigrade Paramacrobiotus experimentalis (Eutardigrada) at Increased Magnesium Perchlorate Levels: Implications for Astrobiological Research
Source: Life (Basel). 2024 Mar 4;14(3):335. doi: 10.3390/life14030335 (PMC10971682; doi:10.3390/life14030335)
Supplement: Supplementary file 1 [file life-14-00335-s001.zip › life-2826648-supplementary.pdf]

Supplementary Material for article

"Long-term survivability of tardigrade Paramacrobiotus experimentalis (Eutardigrada) at increased perchlorate levels: Implications for astrobiological research"

by Paulina Anna Wilanowska, Piotr Rzymski, Łukasz Kaczmarek

Table S1. The body length of the *Pam. experimentalis* specimens in different perchlorate solutions and control at the end of the experiment.

Control [μm]	0.10% [μm]	0.15% [μm]	0.20% [μm]	0.25% [μm]
646.1	490.7	437.3	467.0	367.6
524.6	444.7	469.2	413,2	411.6
558.7	519.3	409.2	404.3	430.7
569.6	482.3	300.7	394.3	374.3
575.6	519.0	381.4	356.3	443.9
761.9	460.4	334.0	317.8	393.3
773.6	463.9	331.0	327.9	
754.6	546.8	457.7		
570.5	396.3	436.7		
749.2	431.2	419.7		
592.7	465.0	461.7		
594.2	454.4	407.6		
746.3	417.5	439.4		
752.0	392.8	412.5		
791.59	482.3			
677.66	488.4			
710.96	466.9			
526.1	422.1			
657.3	420.0			
716.5	526.1			
721.4				
